# Long-Term Follow-Up of Kidney Donors in a Tertiary Care Hospital

**DOI:** 10.7759/cureus.98478

**Published:** 2025-12-04

**Authors:** Poongodi A, Srinivasa Prasad N, Thirumalvalavan Kaliaperumal, Sujith Surendran, Edwin Fernando, Thirumavalavan Subramanian, Murugesan Vellaisamy

**Affiliations:** 1 Nephrology, Government Stanley Medical College and Hospital, Chennai, IND

**Keywords:** follow-up, kidney donors, proteinuria, renal donor clinic, suboptimal compensation

## Abstract

Aim: To estimate the proportion of living kidney donors (completed one year post-donation) who have developed proteinuria, an estimated glomerular filtration rate (eGFR) <60 mL/min/1.73 m², hypertension, or suboptimal renal compensation (defined as <70% of pre-donation glomerular filtration rate (GFR)) during post-donation follow-up. The study also aimed to evaluate the association between donor-related factors and clinical outcomes. To assess the emotional well-being and overall satisfaction of donors through structured, in-person interviews.

Patients and methods: A cross-sectional prospective study design was undertaken to contact all donors of recipients under regular review who had completed at least one year post-donation. Among approximately 250 living renal transplant recipients in active follow-up, about 200 corresponding donors had crossed the one-year post-donation period. Of these, 160 donors responded to the call for follow-up, and 140 completed a comprehensive clinical and laboratory assessment in accordance with the institutional follow-up protocol. Written informed consent was obtained from all participants, and pre-donation records were retrieved for comparative analysis. The emotional well-being and overall satisfaction of donors were assessed through structured, in-person (in formal) interviews. Results were analyzed with IBM SPSS Statistics Software, version 20.0 (IBM Corp., Armonk, New York, USA).

Results: The mean age at donation was 45.4 ± 9.4 years (range: 20-69 years), and the mean age at follow-up was 51.5 ± 9.9 years. The median duration of follow-up was 7 ± 4 years (interquartile range (IQR): 1-23 years), with 30% (n = 42) of donors followed for more than 10 years post-donation. Female donors predominated (77.2%; n = 108), yielding a female-to-male ratio of approximately 3.4:1. Mothers constituted the largest donor subgroup (52.8%; n = 74). Perioperative complications occurred in 8% (n = 7) of donors. Hypertension was noted in 34 (24.3%). Proteinuria (protein-to-creatinine ratio (PCR) > 0.2) was seen in 34 donors (24.3%). Mean eGFR pre- and post-donation was 91.6 ± 16.0 ml/min and 80.3 ± 18.5 ml/min, with a decline of 11 ml/min. One hundred twenty-two donors (87.1%) have eGFR >60 ml/min/1.73 m^2^. Overall, 84.3% (n = 118) achieved optimal and 15.7% (n = 22) showed suboptimal compensation. On multivariate analysis, only suboptimal renal compensation remained an independent predictor of eGFR < 60 ml/min (adjusted OR: 31.43; 95% CI: 7.19-137.2; p < 0.001). Neither donor age nor gender showed a significant association with outcomes such as proteinuria, hypertension, eGFR <60 mL/min/1.73 m^2^,and suboptimal compensation. A vast majority (91%, n = 127) expressed happiness and complete satisfaction with their decision to donate.

Conclusion: In this cohort, female predominance reflected persistent sociocultural patterns. The prevalence of hypertension was comparable to that of the general population. These findings reinforce that modest post-donation changes in proteinuria or eGFR should not discourage donation, given its profound benefits to recipients, donors, and society. Establishing dedicated renal donor clinics for structured, lifelong surveillance is vital to safeguard donor health.

## Introduction

Living-donor renal transplantation provides superior graft and patient survival compared with deceased-donor transplantation [1], making it a cornerstone in managing end-stage renal disease. However, concerns about donor safety persist.

The hyperfiltration theory proposed in the 1980s suggested that adaptive increases in renal blood flow and intraglomerular pressure after nephrectomy could lead to progressive glomerulosclerosis [2]. Since then, multiple studies have evaluated renal and metabolic outcomes in kidney donors, including risks of proteinuria, hypertension, diabetes, and chronic kidney disease (CKD).

Despite extensive global data, evidence from India remains limited. Regional variations in genetics, lifestyle, and healthcare access may influence donor outcomes, highlighting the need for context-specific studies. Moreover, the psychosocial well-being of donors has received little systematic attention.

This cross-sectional prospective study was conducted to determine the proportion of living kidney donors who developed proteinuria, an estimated glomerular filtration rate (eGFR) <60 mL/min/1.73 m², hypertension, or suboptimal renal compensation (defined as <70% of pre-donation glomerular filtration rate (GFR)) during post-donation follow-up. The study also aimed to evaluate the association between donor-related factors, including age and gender, and these clinical outcomes. In addition, donors' emotional well-being and overall satisfaction were assessed through structured, in-person interviews.

## Materials and methods

Study setting and donor recruitment

The first living-donor renal transplantation at our center was performed in April 1986. As of this analysis, 793 living-related renal transplants have been completed. Donor selection followed conventional criteria until 2012, after which the Kidney Disease: Improving Global Outcomes (KDIGO) 2012 Clinical Practice Guideline for the Evaluation and Care of Living Kidney Donors was adopted, with subsequent 2017 updates.

Study design

This study is a cross-sectional prospective study.

Sampling strategy

A structured follow-up initiative was undertaken to contact all living kidney donors whose corresponding recipients remained in regular review and who had completed at least one year post-donation. Among approximately 250 renal transplant recipients in active follow-up, nearly 200 associated donors had surpassed the one-year post-donation period. Recipients were requested to bring their respective donors for evaluation. Of the 160 donors who responded, those with incomplete records were excluded. The final study cohort comprised 140 donors who underwent comprehensive clinical and laboratory assessment as per the institutional follow-up protocol. Written informed consent was obtained from all participants, and pre-donation records were retrieved for comparative analysis.

Clinical and laboratory evaluation

Each renal donor underwent a comprehensive clinical evaluation, including a detailed medical history, assessment of comorbidities, medication use, and measurement of vital parameters. A complete physical examination was performed with specific attention to the cardiovascular and renal systems. Laboratory investigations included hematological and biochemical profiling, serum creatinine, eGFR, fasting blood glucose, lipid profile, and urine analysis, including dipstick testing and spot urine protein-to-creatinine ratio. In addition, all donors underwent renal ultrasonography to assess kidney size, cortical thickness, echotexture, and to screen for any structural abnormalities. These evaluations were conducted in accordance with the institution's standardized post-donation follow-up protocol.

Blood pressure was measured in accordance with KDIGO 2021 recommendations [3]. Two readings were obtained using a standard BP device, and their average was recorded. Hypertension was defined as current use of antihypertensive medication or a mean blood pressure >140/90 mmHg in untreated individuals, as per the 2024 European Society of Cardiology guidelines.

Diabetes was defined as per the American Diabetes Association (ADA) guidelines. Random plasma glucose ≥200 mg/dL (≥11.1 mmol/L) in the presence of classic symptoms of hyperglycemia or documented history of diabetes or current use of anti-diabetic medications at the time of follow-up.

Proteinuria assessment was performed using both urine dipstick testing and the spot urine protein-to-creatinine ratio (PCR). Dipstick-positive results were confirmed with two additional samples, and significant proteinuria was defined as persistence of proteinuria in at least three consecutive samples along with a PCR value >0.2. Renal function was evaluated by measuring serum creatinine levels using the modified Jaffe's method in the institution's biochemistry laboratory. The estimated glomerular filtration rate (eGFR) was calculated using the Chronic Kidney Disease Epidemiology Collaboration (CKD-EPI) 2021 equation, which incorporates serum creatinine, age, and sex to provide a standardized estimate of kidney function.

Renal ultrasonography was performed for all donors as part of the standardized post-donation evaluation. Ultrasound examinations were conducted by experienced radiologists using high-resolution real-time B-mode imaging. Each study included measurement of renal length, cortical thickness, and parenchymal echogenicity of the remaining kidney. The collecting system was assessed for calyceal fullness or hydronephrosis. Ultrasonography was also evaluated for focal lesions, cysts, calculi, or postoperative structural changes.

The emotional well-being and overall satisfaction of donors were assessed through structured, in-person (in formal) interviews. Interview questions were framed by investigators and peer-reviewed before the in-person interview with donors.

Ethical considerations

The study was approved by the Institutional Ethics Committee of Government, Stanley Medical College and Hospital, Chennai (Approval No. ECR/131/Inst/TN/2013/RR-19).

Statistical analysis

Data were analyzed using IBM SPSS Statistics software, version 20.0 (IBM Corp., Armonk, NY, USA). Categorical variables were compared using the chi-square or Fisher's exact test, as appropriate. Continuous variables were summarized using descriptive statistics and compared using the Student's t-test based on data distribution. Correlations between continuous variables were evaluated using Pearson's correlation coefficient. Results are expressed as mean ± standard deviation (SD), and a two-tailed p-value <0.05 was considered statistically significant.

## Results

Donor demographics

The mean age at donation was 45.4 ± 9.4 years (range: 20-69 years), and the mean age at follow-up was 51.5 ± 9.9 years. The median duration of follow-up was 7 ± 4 years (interquartile range (IQR): 1-23 years), with 30 % (n = 42) of donors followed for more than 10 years post-donation. Female donors predominated (77.2 %; n = 108) compared to males (22.8 %; n = 32), yielding a female-to-male ratio of approximately 3.4:1. Mothers constituted the largest donor subgroup (52.8 %; n = 74).

Surgical characteristics

Open donor nephrectomy was performed in 98.6% of donors, reflecting institutional practice during the earlier period of the transplant program. Only two donors (1.4%) underwent laparoscopic nephrectomy. The left kidney was chosen in most cases (81.4%; n = 118) owing to its favorable anatomy and longer renal vein (Table 1). The median hospital stay was seven days (IQR: 5-21 days), and most donors resumed normal activities within three to four weeks of surgery.

**Table 1:** Baseline demographic and clinical characteristics of living kidney donors (n = 140). IQR: interquartile range.

Section	Characteristic	Value/description
General characteristics	Number of donors	140
	Females, n (%)	108 (77.2%)
	Males, n (%)	32 (22.8%)
	Mean age at donation (years)	45.4 (range: 36–54.8)
Age distribution at donation	18–30 years, n (%)	11 (7.8%)
	31–40 years, n (%)	28 (20.0%)
	41–50 years, n (%)	62 (44.2%)
	>50 years, n (%)	39 (27.8%)
Follow-up and duration since donation	Mean age at follow-up (years)	51.5 (range: 41.6–61.4)
	Median post-donation duration (years)	7 ± 4 (IQR: 1–23)
Years since donation	<5 years, n (%)	58 (41.5%)
	5–10 years, n (%)	40 (28.5%)
	>10 years, n (%)	42 (30.0%)
Pre-donation comorbid conditions	Hypertension n (%)	1 (<1%) (on single drug)
	Diabetes mellitus	Nil
	Proteinuria	Nil
	Renal calculi, n (%)	2 (1%)
Donor surgery characteristics	Open nephrectomy, n (%)	138 (98.6%)
	Laparoscopic nephrectomy, n (%)	2 (1.4%)
	Side of nephrectomy	Left: 114 (81.4%); right: 26 (18.6%)
	Median hospital stay (days)	7 (IQR: 5–21)
Perioperative complications	Total complications	7 (5.0%)
	Lung collapse, n (%)	1 (<1%)
	Hydropneumothorax, n (%)	1 (<1%)
	Surgical site infection, n (%)	5 (3.0%)
	Incisional hernia, n (%)	8 (5.0%)

Perioperative complications

Perioperative complications occurred in 8 % (n = 7) of donors. Documented events included lung collapse (n = 1), pneumothorax (n = 1), and surgical-site infection (stitch abscess, n = 5). The donor with pneumothorax required intercostal drain insertion and recovered fully without sequelae. Late complications were observed in five donors, all presenting with incisional hernia; three underwent mesh repair with favorable outcomes.

Proteinuria

At follow-up, 24.3% (n = 34) of donors had detectable proteinuria, measured by spot urine protein-to-creatinine ratio (PCR). Among them, 19 donors (13.6%) had mild proteinuria (PCR 0.2-0.5), 10 donors (7.2%) had moderate proteinuria (>0.5 to <1.0), and five donors (3.5%) exhibited significant proteinuria (≥1.0) (Table 2).

**Table 2:** Distribution of donors based on urine protein-to-creatinine ratio (PCR) at follow-up. PCR: protein-to-creatinine ratio, urine PCR >0.2 is considered significant.

Urine PCR (mg/mg)	Frequency N (%)
0.2–0.5	19 (13.6%)
>0.5 to <1.0	10 (7.2%)
≥1.0	5 (3.5%)
Total (≥0.2)	34 (24.3%)

On univariate analysis, age at donation (p = 0.01), diabetes (p = 0.016), and suboptimal compensation (p = 0.012) showed significant associations with post-donation proteinuria. Age at follow-up (p = 0.23) and post-donation GFR < 60 ml/min (p = 0.135) showed only modest trends. After adjustment (Table 3), none of the variables remained statistically significant. The adjusted model indicated no independent association between proteinuria and age at donation (OR = 0.972, p = 0.570), age at follow-up (OR = 1.059, p = 0.236), GFR < 60 (OR = 2.946, p = 0.135), suboptimal compensation (OR = 1.523, p = 0.514), or diabetes (OR = 2.588, p = 0.81). Thus, the crude associations observed were not retained after adjustment, suggesting the influence of confounding factors.

**Table 3:** Multivariate analysis for proteinuria. The statistical method used is multivariate logistic regression. GFR: glomerular filtration rate; OR > 1: increased odds of proteinuria; OR < 1: decreased odds of proteinuria; p < 0.05: statistically significant. The p-value was calculated using the chi-square test.

Variables	Univariate P-value	Adjusted OR	P < 0.05
Age at donation	0.01	0.972 (0.881-1.072)	0.570
Age at follow-up	0.23	1.059 (0.963-1.163)	0.236
Post-donation GFR <60 ml/min	0.135	2.946 (0.713-12.168)	0.135
Suboptimal compensation	0.012	1.523 (0.430-5.379)	0.514
Diabetes	0.0161	2.588 (0.890-7.528)	0.81
Constant		0.039	0.11

Hypertension

Mean systolic BP increased slightly from 120 ± 10.2 mmHg pre-donation to 124.9 ± 19.7 mmHg post-donation (p = 0.205) (Table 4). Hypertension was observed in 24.3% (n = 34) of donors, all with well-controlled BP on ≤2 agents, mainly calcium channel blockers and β-blockers. Prevalence was similar between men and women (26.7% vs. 23.6%; p = 0.81) and showed no significant association with family history, proteinuria, or duration since donation. Donors aged >45 years had a higher prevalence than those <45 years (31.3% vs. 14%; p = 0.019). CKD incidence did not differ by hypertensive status (p = 0.55). Multivariate analysis indicated a non-significant trend toward higher hypertension risk in donors who developed diabetes (p ≈ 0.05) (Table 5).

**Table 4:** Comparison of pre- and post-donation variables among renal donors. eGFR: estimated glomerular filtration rate; PCR: protein-to-creatinine ratio; BP: blood pressure; NA: not applicable; p < 0.05: statistically significant.

Variables	Pre-donation	Post-donation	P (<0.05)
Mean serum creatinine (mg/dL) (mean ± SD)	0.86 ± 0.12	0.97 ± 0.24	<0.0001
eGFR (CKD-EPI) (mL/min/1.73 m²) (mean ± SD)	91.6 ± 16.0	80.3 ± 18.5	<0.001
Urine PCR ≥ 0.2 n (%)	Nil	34 (24.3%)	NA
Hypertension n (%)	Nil	34 (24.3%)	0.205
Systolic BP (mm Hg) (mean ± SD)	120 ± 10.2	124.9 ± 19.7	0.205
Diabetes n (%)	Nil	24 (17.1%)	NA
eGFR < 60 mL/min n (%)	Nil	18 (12.9%)	NA
45–60 mL/min	–	14 (10.1%)	NA
30–44 mL/min	–	3 (2.1%)	NA
15–29 mL/min	–	1 (0.7%)	NA
<15 mL/min	–	Nil	NA
Mean hemoglobin (g/dL) (mean ± SD)	12.6 ± 1.4	12.16 ± 1.5	NA
Mean kidney length (cm) (mean ± SD)	9.6 ± 0.77	10.3 ± 0.93	<0.0001

**Table 5:** Multivariate analysis for hypertension. Statistical significance was set at p < 0.05. The p-value was calculated using the chi-square test.

Variables	Adjusted OR	P-value
Age at donation	1.040 (0.948-1.042)	0.406
Age at follow-up	1.010 (0.919-1.111)	0.830
Diabetes	2.499 (0.930-6.718)	0.069
Constant	0.021	0.002

Diabetes

Prevalence of diabetes in donor's post-donation is 17.1 % (N = 24). None of the donation-related factors showed an association with diabetes in multivariate analysis (Table 4).

Suboptimal compensation

Post-donation renal compensation was categorized as optimal (>70% of pre-donation eGFR) or suboptimal (<70% of pre-donation eGFR). Overall, 84.3% (n = 118) achieved optimal and 15.7% (n = 22) showed suboptimal compensation. On multivariate analysis, higher pre-donation eGFR significantly predicted suboptimal compensation (adjusted OR: 1.14; 95% CI: 1.03-1.26; p = 0.011). Post-donation CKD (eGFR <60 mL/min) showed the strongest association (adjusted OR: 443.47; 95% CI: 29.16-6744.80; p < 0.001). Diabetes also increased risk (adjusted OR: 17.41; 95% CI: 2.26-134.20; p = 0.006). Gender, post-donation proteinuria, and serum creatinine were not significantly associated (Table 6).

**Table 6:** Multivariate analysis for suboptimal compensation. PCR: protein-to-creatinine ratio, eGFR: estimated glomerular filtration rate, CKD: chronic kidney disease. The table presents results from a multivariate logistic regression model to identify independent predictors of suboptimal compensation while adjusting for multiple variables simultaneously. The p-value was calculated using the chi-square test.

Variables	Adjusted OR (CI)	P < 0.05
Gender	1.015 (0.106-9.714)	0.990
Post-PCR	0.542(0.089-3.291)	0.505
Serum creatinine	1.049 (0.000-39093.334)	0.993
eGFR (ml/min) Pre-donation	1.140 (1.030-1.261)	0.011
CKD	443.473 (29.159-6744.802)	0.000
Diabetes	17.408 (2.258-134.196)	0.006
Constant	0.000	0.073

Post-donation renal function (estimated glomerular filtration rate (eGFR) <60 ml/min)

On univariate analysis, lower eGFR was significantly associated with male gender (p = 0.034), suboptimal renal compensation (p < 0.001), proteinuria (p = 0.012), and higher age at both donation and follow-up (p = 0.001). Duration since donation showed no significant relationship (p = 0.290). On multivariate analysis, only suboptimal renal compensation remained an independent predictor of eGFR <60 ml/min (adjusted OR: 31.43; 95% CI: 7.19-137.2; p < 0.001) (Table 7).

**Table 7:** Multivariate analysis for eGFR < 60 ml/min. The constant term is 0.00 with a p-value of 0.000, indicating the model's intercept. eGFR: estimated glomerular filtration rate. The p-value was calculated using the chi-square test.

Variables	Adjusted OR	p < 0.05
Age at follow-up	1.005 (0.884-1.144)	0.934
Age at donation	1.129 (0.973-1.308)	0.104
Post-donation proteinuria	1.720 (0.390-7.581)	0.473
Suboptimal compensation	31.43 (7.197-137.2)	0.000
Gender	1.239 (0.278-5.535)	0.779
Constant	0.00	0.000

Emotional aspects of kidney donors

Assessment of donor awareness and emotional well-being revealed a predominantly positive outlook. More than half of the donors (56.6%, n = 79) were aware of the need for regular post-donation follow-up. A vast majority (91%, n = 127) expressed happiness and complete satisfaction with their decision to donate, while only 6.1% reported regret or unhappiness. Most donors (76.8%, n = 107) were unconcerned about future renal health risks, and 88.9% (n = 124) actively encouraged others to consider kidney donation, reflecting strong psychological well-being and altruistic motivation post-donation.

## Discussion

The demographic profile of our donor population aligns closely with previously published Indian and international data.

Gender disparity

A marked female predominance (77.2%, n = 108) was observed among donors, consistent with other Indian studies. Muthusethupathi et al. [4] similarly reported that females constituted two-thirds (64%) of donors, while 84% of recipients were male--reflecting persistent sociocultural influences driving donation decisions within families. In contrast, international registries such as the US Organ Procurement and Transplantation Network (US OPTN) and European cohorts report a more balanced gender distribution (55-60% female). Despite advancements in awareness and policy, gender disparity in India appears largely unchanged over the past 25 years.

Age distribution and follow-up

Most donors were aged 41-50 years (44.2%, n = 62), followed by those >50 years (27.8%, n = 39), indicating that nearly three-fourths were over 40 years at the time of donation. Only a small proportion (7.8%, n = 11) were below 30 years, aligning with standard donor selection practices that favor medically stable, middle-aged adults. Notably, 30% (n = 42) had more than 10 years of follow-up, lending strength to the reliability of long-term outcome assessment in this cohort.

Baseline health and surgical profile

Pre-donation comorbidities were infrequent, reflecting stringent selection criteria and adherence to modern donor safety standards. Open nephrectomy remained the predominant surgical technique (98.6%), with a low perioperative complication rate (5%, n = 7)-significantly below that reported in comparable series (16.8%) [5,6]. This underscores the procedural safety and high-quality perioperative care at our center.

Proteinuria

In our study group, no single clinical variable independently predicted the occurrence of proteinuria post-donation, suggesting preserved renal safety over long-term follow-up. Similar findings were reported by Ibrahim et al. [7], who observed post-donation proteinuria in 12.7% of donors without significant association with baseline age or gender after adjustment for confounding factors.

Post-donation hypertension

Post-donation hypertension was observed in 24.3% of our donors, consistent with previous reports (20-30%) [7]. Older donor age emerged as the main determinant, while gender, time since donation, and proteinuria were not significant predictors. Importantly, hypertensive donors did not show increased risk of CKD, suggesting that kidney donation does not exacerbate hypertensive renal injury when blood pressure is well controlled.

Comparison with published donor cohorts

Compared with pooled data from Garg et al. [8] (Table 8), our cohort (n = 140) showed broadly similar post-donation outcomes. Median follow-up was comparable (seven vs. six years), supporting an adequate observation period. Donors in our study were slightly older (45.4 vs. 41 years) with lower pre-donation eGFR (91.6 ± 16.0 vs. 111 mL/min), yet post-donation renal function remained well preserved (mean eGFR 80.3 ± 18.5 vs. 86 ± 19 mL/min). Serum creatinine rose mildly (0.86 → 0.97 mg/dL), and blood pressure remained stable, mirroring global trends. The proportion of donors with eGFR <60 mL/min was similar to international estimates (12.9% vs. 12.2%), and no donor progressed to end-stage renal disease. Despite lower baseline renal function and slightly older age, Indian donors in our study demonstrated a post-donation trajectory comparable to international cohorts, highlighting the safety and resilience of carefully selected living kidney donors.

**Table 8:** Comparison of renal outcomes in kidney donors: pooled analysis (48 studies; n = 5048) vs. our cohort (n = 140). Values are expressed as mean ± SD, median (IQR), or percentage, as appropriate. eGFR was calculated using the CKD-EPI 2021 equation in the present study. Post-donation GFR categories were classified as per KDIGO guidelines. Meta-analysis by Garg et al [8]. eGFR: estimated glomerular filtration rate; IQR: interquartile range; KDIGO: Kidney Disease: Improving Global Outcomes.

Variables	Forty-eight studies (5048 donors) [8]	Our study (140 donors)
Median years of donation in years (IQR)	6 (1-25)	7 (1-23)
Average age of donors in years (IQR)	41 (26-59)	45.4 (36-54.8)
Mean serum creatinine (mg/dl) (pre-donation)	0.92 (0.58-1.13)	0.86 ± 0.12
Mean eGFR ml/min (pre-donation)	111 (91-132)	91.6 ± 16.0
Mean serum creatinine (mg/dl) (post-donation)	1.11 (1.04-2.14)	0.97 ± 0.24
Mean eGFR ml/min (post-donation)	86 ± 19	80.3 ± 18.5
Average systolic blood pressure (mm Hg) (pre-donation)	121 ± 11	119.97 ± 10.21
Average systolic blood pressure (mm Hg) (post-donation)	124.3 ± 19.65	124.9 ± 19.65
eGFR category	Percentage	Percentage (frequency)
<60 ml/min	12.2%	12.9% (n = 18)
30-59 ml/min	12%	12.2% (n = 17)
15-29 ml/min	0.2%	0.7% (n = 1)
<15 ml/min	None	None

Comparison with the Indian donor study

Compared with the cohort reported by Sahay et al. [9] (Table 9), our donors had a comparable follow-up duration (7 years vs. 63 months) but were slightly older at donation (45.4 vs. 41.3 years) and exhibited lower pre-donation eGFR (91.6 ± 16.0 vs. 102.7 ± 6.9 mL/min). Despite this, post-donation renal function was marginally better in our cohort (80.3 ± 18.5 vs. 74.5 ± 14.6 mL/min), reflecting adequate compensatory adaptation. The mean decline in eGFR was smaller in our donors (11 vs. 26 mL/min), suggesting better long-term preservation of renal function. Hypertension prevalence was lower (24.3% vs. 46%), while proteinuria was more frequent (24.3% vs. 14%), likely attributable to the lower PCR threshold used in our study (0.2 vs. 0.3).

**Table 9:** Comparison of demographic and post-donation clinical characteristics of kidney donors in the present study with those reported by Sahay et al. Values are expressed as mean ± SD or median (range). Estimated glomerular filtration rate (eGFR) was calculated using the CKD-EPI 2021 equation. Proteinuria was defined as urine protein-to-creatinine ratio (PCR) >0.2 in the present study and >0.3 in the study by Sahay et al. [9].

Variables	Sahay et al. [9]	Our study
Median duration post-donation (N)	63 (3-264) months	7 (1-23) years
Female donors (%)	56%	78%
Mean age at donation (years)	41.26 ± 8.12	45.4 (36-54.8)
Mean serum creatinine (mg/dl) (pre-donation) (mean ± SD)	0.97 ± 0.09	0.86 ± 0.12
Mean serum creatinine (mg/dl) (post-donation) (mean ± SD)	1.22 ± 0.82	0.97 ± 0.24
Average systolic blood pressure (mm Hg) pre-donation	119.8 ± 6.1	119.97 ± 10.21
Prevalence of hypertension (%)	46%	24.3%
Average systolic blood pressure (mm Hg) post-donation	129.76 ± 13.84	124.9 ± 19.65
Mean eGFR ml/min (pre-donation) (mean ± SD)	102.7 ± 6.9	91.6 ± 16.0
Mean eGFR ml/min) (post-donation) (mean ± SD)	74.54 ± 14.64	80.3 ± 18.5
Incidence of proteinuria (%)	14% (PCR > 0.3)	24.3% (PCR > 0.2)
Mean length of kidney (cm) (pre vs. post) (mean ± SD)	9.46 ± 0.39 vs. 10.60 ± 0.73	9.6 ± 0.77 vs. 10.3 ± 0.93

In our study, higher pre-donation eGFR modestly increased the risk of suboptimal compensation (14% per unit), while post-donation CKD and diabetes were strong independent predictors (443-fold and 17-fold higher odds, respectively). Gender and post-donation proteinuria were not significant, aligning with Taner et al., who reported similar compensatory gains in medically complex and standard donors five years post-donation [10].

Ibrahim et al. reported that 15% of donors developing measured GFR <60 ml/min after ~40 years, comparable to our findings [7]. Post-donation GFR and low-grade proteinuria should be interpreted cautiously. eGFR may underestimate true kidney function in donors [11], and conventional CKD cut-offs may not suit healthy Indian adults [12]. In our cohort, 10.5% were labeled CKD stage 3 despite being clinically well. Proteinuria likely reflects adaptive hyperfiltration rather than disease, though its long-term impact on renal decline remains uncertain [13,14]. Donation fosters long-term psychological well-being, minimal regret, and sustained altruism when supported with proper counseling and follow-up.

Renal donor clinic

Our findings highlight the urgent need for dedicated renal donor clinics to ensure systematic, lifelong follow-up of kidney donors. Nearly 90% of donors in our cohort were not under regular surveillance, primarily due to limited awareness and logistical challenges. Establishing such clinics would facilitate early detection and management of age-related comorbidities, support preventive care, and enable the collection of robust national-level data on long-term donor outcomes. This is particularly relevant in the Indian context, where donors often present with lower pre-donation eGFR, higher prevalence of hypertension and proteinuria, and a marked gender disparity, underscoring the need for region-specific policies and structured donor registries.

Limitations

This study has several limitations. Renal function was estimated using CKD-EPI 2021 rather than measured GFR, which may underestimate true function in healthy donors. The absence of age- and gender-matched controls precludes distinguishing donation-related changes from those due to aging or comorbidities. Selection bias and external applicability warrant further consideration. Because participation relied on donors responding to a structured follow-up call, those who returned for evaluation may differ systematically from non-responders in health status, motivation, or access to care. This could lead to an overrepresentation of healthier or more health-conscious donors and may underestimate adverse outcomes. Additionally, as the study was conducted within a single center with its own donor selection practices, follow-up protocols, and sociodemographic profile, the findings may not be fully generalizable to other transplant programs. Variations in donor evaluation criteria, long-term monitoring, and population characteristics across institutions and regions could limit the external applicability of the results. Acknowledging these factors is important when interpreting the study's conclusions and comparing them with broader donor outcome data.

## Conclusions

When carefully selected and monitored, living kidney donation continues to be a safe and ethically sound practice. In this cohort, female predominance likely reflects prevailing sociocultural patterns, and perioperative complications were minimal. The prevalence of hypertension was comparable to that of the general population, and mild proteinuria did not translate into an increased risk of CKD or functional decline. However, these findings should be interpreted in light of the study's limitations. The possibility of selection bias exists, as donors who presented for follow-up may differ from non-responders in health status or motivation. Additionally, the single-center design and institution-specific practices may limit the external applicability of the results to broader donor populations. Despite these constraints, the study underscores that modest post-donation changes in proteinuria or eGFR should not discourage donation, given the substantial benefits conferred to recipients, donors, and society. Establishing dedicated renal donor clinics to ensure structured, lifelong surveillance remains essential. Future multicentric studies with appropriately matched controls, including sibling comparators to account for shared genetic and environmental risk, are warranted to better characterize long-term donor outcomes.

## References

[1] Nemati E, Einollahi B, Lesan Pezeshki M, Porfarziani V, Fattahi MR (2014). Does kidney transplantation with deceased or living donor affect graft survival?. Nephrourol Mon.

[2] Brenner BM, Meyer TW, Hostetter TH (1982). Dietary protein intake and the progressive nature of kidney disease: the role of hemodynamically mediated glomerular injury in the pathogenesis of progressive glomerular sclerosis in aging, renal ablation, and intrinsic renal disease. N Engl J Med.

[3] Cheung AK, Chang TI, Cushman WC (2021). Executive summary of the KDIGO 2021 clinical practice guideline for the management of blood pressure in chronic kidney disease. Kidney Int.

[4] Muthusethupathi MA, Rajendran S, Jayakumar M, Vijayakumar R (1998). Evaluation and selection of living related kidney donors--our experience in a government hospital. J Assoc Physicians India.

[5] Lentine KL, Lam NN, Axelrod D (2016). Perioperative complications after living kidney donation: a national study. Am J Transplant.

[6] Dagnæs-Hansen J, Kristensen GH, Rohrsted M, Sørensen SS, Røder A (2025). Early and late surgical complications following living donor nephrectomy. Scand J Urol.

[7] Ibrahim HN, Foley R, Tan L (2009). Long-term consequences of kidney donation. N Engl J Med.

[8] Garg AX, Muirhead N, Knoll G (2006). Proteinuria and reduced kidney function in living kidney donors: a systematic review, meta-analysis, and meta-regression. Kidney Int.

[9] Sahay M, Narayen G (2007). Risk of live kidney donation--Indian perspective. J Assoc Physicians India.

[10] Taner T, Iqbal CW, Textor SC, Stegall MD, Ishitani MB (2015). Compensatory hypertrophy of the remaining kidney in medically complex living kidney donors over the long term. Transplantation.

[11] Tent H, Rook M, Stevens LA (2010). Renal function equations before and after living kidney donation: a within-individual comparison of performance at different levels of renal function. Clin J Am Soc Nephrol.

[12] Yadav AK, Ghosh A, Kumar V (2024). Development and validation of an accurate creatinine-based equation to estimate glomerular filtration rate for the adult Indian population: design and methods. Indian J Nephrol.

[13] Hostetter TH, Olson JL, Rennke HG, Venkatachalam MA, Brenner BM (1981). Hyperfiltration in remnant nephrons: a potentially adverse response to renal ablation. Am J Physiol.

[14] Lenihan CR, Busque S, Derby G, Blouch K, Myers BD, Tan JC (2015). Longitudinal study of living kidney donor glomerular dynamics after nephrectomy. J Clin Invest.

